# Wind conditions and geography shape the first outbound migration of juvenile honey buzzards and their distribution across sub-Saharan Africa

**DOI:** 10.1098/rspb.2017.0387

**Published:** 2017-05-24

**Authors:** W. M. G. Vansteelant, J. Kekkonen, P. Byholm

**Affiliations:** 1 Institute for Biodiversity and Ecosystem Dynamics, University of Amsterdam, PO Box 94248, 1090 GE Amsterdam, The Netherlands; 2 Vansteelant Eco Research, Dijkgraaf 35, 6721 NJ Bennekom, The Netherlands; 3 Department of Biosciences, University of Helsinki, PO Box 65, 00014 Helsinki, Finland; 4 Bioeconomy Research Team, Novia University of Applied Sciences, 10600 Ekenäs, Finland

**Keywords:** bird migration, orientation, weather, behavioural development, satellite-tracking

## Abstract

Contemporary tracking studies reveal that low migratory connectivity between breeding and non-breeding ranges is common in migrant landbirds. It is unclear, however, how internal factors and early-life experiences of individual migrants shape the development of their migration routes and concomitant population-level non-breeding distributions. Stochastic wind conditions and geography may determine whether and where migrants end up by the end of their journey. We tested this hypothesis by satellite-tagging 31 fledgling honey buzzards *Pernis apivorus* from southern Finland and used a global atmospheric reanalysis model to estimate the wind conditions they encountered on their first outbound migration. Migration routes diverged rapidly upon departure and the birds eventually spread out across 3340 km of longitude. Using linear regression models, we show that the birds' longitudinal speeds were strongly affected by zonal wind speed, and negatively affected by latitudinal wind, with significant but minor differences between individuals. Eventually, 49% of variability in the birds' total longitudinal displacements was accounted for by wind conditions on migration. Some birds circumvented the Baltic Sea via Scandinavia or engaged in unusual downwind movements over the Mediterranean, which also affected the longitude at which these individuals arrived in sub-Saharan Africa. To understand why adult migrants use the migration routes and non-breeding sites they use, we must take into account the way in which wind conditions moulded their very first journeys. Our results present some of the first evidence into the mechanisms through which low migratory connectivity emerges.

## 1. Introduction

One of the most robust patterns emerging from contemporary tracking studies of migrant landbirds is one of low migratory connectivity, whereby individuals which breed in close vicinity of each other diverge across huge geographical distances during the non-breeding phase of their annual cycle [1]. Although there are exceptions where strong connectivity between breeding and non-breeding sites exists [2,3], in most migrant landbirds, individuals from different breeding populations are likely to mix during the non-breeding season. We also know that migrant landbirds are typically highly faithful to individual breeding and non-breeding sites, temporarily residing in two or more areas along their individual migration cycle [4]. It remains unclear, however, how innate and environmental factors affect the development of individual migration routines during early life [5]. A major challenge in this regard is to resolve how stochastic environmental influences shape the first outbound migration of juvenile migrants [4]. Although many migrant landbirds travel in mixed-age groups, juveniles migrate independently from their parents and other elders in numerous species [6,7]. Such unexperienced migrants are assumed to follow an innate migratory heading for a predetermined amount of time during one or more bouts of migratory flight [8,9], which explains why young and inexperienced migrants are often observed not to compensate for wind drift [10–13] or experimentally induced displacements [14], and which suggests they only manage to settle wintering territories if they do not drift too far from suitable habitat. In such a system, there is a great potential for environmental factors, and especially geography and atmospheric circulation patterns, to influence the distribution of juvenile migrants, moulding patterns of migratory connectivity within and among breeding populations [1,4,15]. Much of this theory, however, have been developed based on site-specific radar observations and experiments, and still needs to be tested by tracking juvenile migrants [7,16].

Very little is known about how environmental factors shape the first outbound migrations of juvenile migrant landbirds that rely on genetic information because most juvenile tracking studies so far have been conducted on large species such as storks [17–19], cranes [20], kites [16], eagles [21,22] and vultures [23]; all of which learn strategic migration routes and stop-over sites from elder conspecifics. Only a handful of studies have tracked juvenile migrants that travel independently from elders, and these often yielded contrasting results about the role of innate and environmental factors in the development of individual migration routines. A satellite-tracking study of juvenile ospreys *Pandion haliaetus* and honey buzzards *Pernis apivorus* from Scandinavia, for example, confirmed that juveniles did not compensate for sidewinds towards predetermined goals as their adult conspecifics did [24,25]. By contrast, juveniles of other migrant landbird species are capable of navigating towards targeted non-breeding areas without elder guidance. Juvenile Eleonora's falcons *Falco eleonora* [26] and juvenile common cuckoos *Cuculus canorus* [27], for example, independently navigate to restricted non-breeding ranges, respectively, on Madagascar and south of the Congo Basin. Juvenile Eleonora's falcons from Sardinia thereby used a complex route involving a major shift in migratory orientation after they crossed the Sahara [26], whereas a juvenile common cuckoo followed a remarkably straight path from northern Europe to northern Angola [27]. Some juvenile waders engage in long and complex but adaptive detours that appear to be programmed genetically [28,29]. Contrary to expectation, it has also been shown that juvenile songbirds can compensate for large geographical displacements from their ‘normal’ migratory route [30] and that complex migration routes can be genetically hard-wired [31]. Juvenile migrants may also engage in remarkably straight trans-oceanic autumn migrations, which may require a more sophisticated strategy than simple vector-based navigation [32]. There is, in conclusion, still little empirical information about the influence of environmental conditions on the orientation of juvenile migrants and the role of early-life experiences in shaping individual migration routines and migratory connectivity [1,4,33].

In this paper, we present the first results of an ongoing study into the ontogeny of individual migration routines of European honey buzzards. Between 2011 and 2014, thirty one fledgling honey buzzards from southern Finland were equipped with Argos tracking devices and GSM-GPS-trackers before they left the nest. Three fledglings were confirmed to have died on the nest owing to predation (two) and sickness (one) and one died in Estonia shortly upon departure (electronic supplementary material, table S1) so we obtained a good sample of tracking data for 27 juveniles. Although adult honey buzzards engage in complex detours to circumvent geographical barriers and to exploit predictable large-scale wind regimes [34], the juveniles are unable to learn these routes during their first migration because they initiate migration one to two weeks later than the adults [24]. The juveniles then seem to follow an innate migratory heading [25], as expected for the bulk of migrant landbird species, and they do not circumvent geographical barriers in the same way as larger soaring migrants do [21,23,35]. As a result, and as juvenile honey buzzards do not compensate for side winds [10], we expect stochasticity in wind conditions on migration to determine at what longitude these juvenile migrants settle in sub-Saharan Africa. We test this hypothesis by annotating tracking data with wind estimates from a global atmospheric reanalysis model [36–38] and modelling the birds' longitudinal speed in relation to wind conditions encountered en route, accounting for possible individual differences in orientation. We also map residual (i.e. predicted–observed) longitudinal speeds to identify during what parts of the journey birds moved westward or eastward faster than predicted. Some birds may, for example, orient downwind more over sea than over land, in which case we expect to see higher residual longitudinal speeds over the Baltic and the Mediterranean for those individuals.

## 2. Methods

### (a) Origin and tracking of juvenile honey buzzards

As a part of a long-term study [39,40], 21 honey buzzard nests on 16 territories in southern Finland (latitude 61°14′–63°12′ N, longitude 21°16′–23°31′ E) were visited between 2011 and 2014. Typically, nests were visited once in June to determine occupied nests and again in mid-July to ring chicks. A third nest visit was timed to the final stage of the brood phase to equip fledglings with solar-powered Argos GPS platform terminal transmitters (PTTs) (Microwave Telemetry Inc.) or GSM-GPS-trackers (Microwave Telemetry Inc., Ecotone). Tags weighed 22–27 g corresponding to approximately 3% of the birds' body mass at the time of deployment (913 ± 82 g; avg ± s.d., *n* = 31). We used the body-loop attachment method with a Teflon ribbon harness [41]. The amount and type of data the PTTs/trackers delivered varied depending on tracker model and programming schedule, but also other factors such as weather (cf. [42]).

The sex of the nestlings (17 females and 14 males, electronic supplementary material, table S1) was determined from DNA as extracted from blood samples using the salt extraction method. Introns of the sex-chromosome linked CHD gene were amplified to distinguish the sexes [43]. Ten microlitres of PCR reaction contained 5 µl of Phusion master mix (Thermofisher Scientific), 10 pmol of primers 2550F and 2718R, 2 µl of dH_2_O and 1 µl of DNA extract. The PCR products were separated on a 2% agarose gel.

### (b) Data preparation

Honey buzzards engage in pre-migratory movements in Europe and also make itinerant movements within sub-Saharan Africa during the non-breeding season. We therefore developed some simple rules on the basis of which to categorize the migration period and checked whether the endpoints we calculated were representative of the longitude at which honey buzzards settled in sub-Saharan Africa (see the electronic supplementary material, figure S1 for full explanation).

Because honey buzzards interrupt travel at night, and possibly under adverse weather conditions, we excluded all resting events from our analyses. We did this by removing all overland locations where ground speed was lower than 1.39 m s^−1^ (approx. 5 km h^−1^). We did not exclude any fixes above sea, except for the fixes of M4 in the night of 3–4 October 2013 because this bird roosted on a ship. We calculated the loxodromic distance and time interval from each location to the next to determine the birds' ground speeds and used vector trigonometry to determine the longitudinal component of the birds' ground speeds (i.e. westward/eastward speed, *U*_bird_). We determined whether fixes were situated over land or over water using the Global Self-consistent Hierarchical High-resolution Shoreline Database [44].

### (c) Influence of wind on hourly longitudinal bird speed

Using the RNCEP package [36], we annotated every fix with zonal (i.e. westward(−)/eastward(+), *U*_wind_) and latitudinal (i.e. southward(−)/northward(+), *V*_wind_) wind components by linearly interpolating wind data from the 925 mB pressure level (corresponding to an average flight altitude of approx. 700 m [37]) in the NCEP global atmospheric reanalysis model [38]. Reanalysis data are generated on a 2.5° × 2.5° grid four times daily and resolve large-scale circulation patterns that can be used reliably to estimate wind conditions at the altitude of flight of soaring raptors [34,45,46]. Summary statistics for *U*_bird_, *U*_wind_ and *V*_wind_ for each of the 27 birds that were used in this analysis are provided in the electronic supplementary material, table S2.

We then constructed generalized linear regression models (GLMs) to determine how hourly longitudinal bird speed (*U*_bird_) was affected by *U*_wind_ and *V*_wind_. We constructed a model including only *U*_wind_, a model including the additive effects of *U*_wind_ and *V*_wind_, and a model including an interaction effect between *U*_wind_ and *V*_wind_. We then selected the most parsimonious model based on Akaike's information criterion (AIC [47]).

### (d) Innate and parental factors

Linear regression plots show a positive relationship between *U*_bird_ and *U*_wind_ for all individuals, regardless of sex or territory where they hatched (electronic supplementary material, figure S2). Some individuals do seem to move west or east in windless conditions faster than others. We therefore extended the most parsimonious GLM with randomly varying intercepts per individual [48]. Fledglings tagged on the same territory may behave similarly owing to parental effects. We therefore constructed a mixed linear effects model with a nested design to allow for randomly varying intercepts between individuals and territories. To identify the most parsimonious model for *U*_bird_, we then compare AIC and log-likelihood values using a restricted maximum likelihood approach [49].

### (e) Identifying influential geographical features

Several factors such as geography, topography, thermal soaring conditions and time of day may influence the rate at which birds move longitudinally. However, instead of running an exhaustive model selection procedure with possibly confounding predictor variables, we decided to map residual (i.e. observed–predicted) hourly longitudinal speeds based on the most parsimonious mixed effects model. This allows us to visualize where birds responded differently to wind than they do on average across the entire flyway.

### (f) Influence of wind and geography on the total longitudinal displacement of birds

Out of 27 birds that departed from Finland, 24 ultimately survived their first migration and 23 of those yielded sufficient data to quantify wind conditions along their entire trip (electronic supplementary material, table S1). We calculated the total longitudinal displacement (Δ_long_[°]) of these 23 individuals by subtracting the longitude at which the birds started migration from the longitude at which they ended migration (electronic supplementary material, table S3). We then constructed multiple linear regression models to predict the birds' total longitudinal displacements as a function of the mean zonal wind (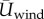) and the mean latitudinal wind (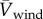) encountered en route.

The birds diverge rapidly upon departure from Finland, partly because one-third of all birds departs via Scandinavia. We also constructed two models including an additional categorical variable to account for this route choice and selected the most parsimonious model including only significant predictor variables based on AIC [47].

Mixed effect models were implemented using the lmer-package in 64-bit R v. 3.3.3. All maps were produced using the ggplot2-package [49]. The three-dimensional plot (electronic supplementary material, figure S3*a*) was produced with the rgl-package [50].

## 3. Results

Our tracking data revealed a rapid divergence of juvenile migration routes upon departure from Finland, whereby birds spread out across 1034 km longitude by the time they reached latitude 55° N at the southern end of the Baltic Sea. Just before the Mediterranean, the spread had increased to 2286 km and by the end of migration, individuals had spread out across 3340 km longitude.

### (a) Influence of wind conditions on hourly longitudinal bird speed

Linear regression models confirmed that *U*_wind_, and to a lesser extent *V*_wind_, significantly affected *U*_bird_. Our most parsimonious model (table 1, model 3; electronic supplementary material, figure S3*a*) shows that on average juvenile honey buzzards moved westward at a rate of 0.76 m s^−1^ in the absence of winds, and that they drifted approximately 0.5 m s^−1^ with every 1 m s^−1^ increase of *U*_bird_ in either direction. In addition, there was a significant negative effect of *V*_wind_ and a significant negative interaction effect of *U*_wind_ : *V*_wind_ on *U*_bird_ (table 1, model 3), indicating that the degree to which birds drifted with zonal winds was exacerbated by headwinds (i.e. northward winds, *V*_wind_ > 0).

**Table 1.** Statistical summary of multiple linear regression models predicting hourly longitudinal bird speed (*U*_bird_) as a function of zonal (*U*_wind_) and latitudinal (*V*_wind_) wind components encountered en route. (Intercepts estimate the mean *U*_bird_ in the absence of wind. The most parsimonious model is given in italics.)

	intercept		*β_U_*_wind_		*β_V_*_wind_		*β_U_*_wind__:_ *_V_*_wind_		AIC	adjusted *R*^2^
	estimate	*p*-value	estimate	*p*-value	estimate	*p*-value	estimate	*p*-value		
model 1	−0.63	3.02 × 10^−20^	0.53	3.46 × 10^−245^					13478.50	0.36
model 2	−0.76	4.44 × 10^−28^	0.52	3.11 × 10^−243^	−0.15	1.55 × 10^−20^			13394.09	0.38
*model 3*	*−0.76*	*5.67 × 10^−29^*	*0.47*	*1.13 × 10^−187^*	*−0.11*	*4.83 × 10^−11^*	*−0.03*	*1.17 × 10^−9^*	*13313.64*	*0.40*

**Figure 1.**
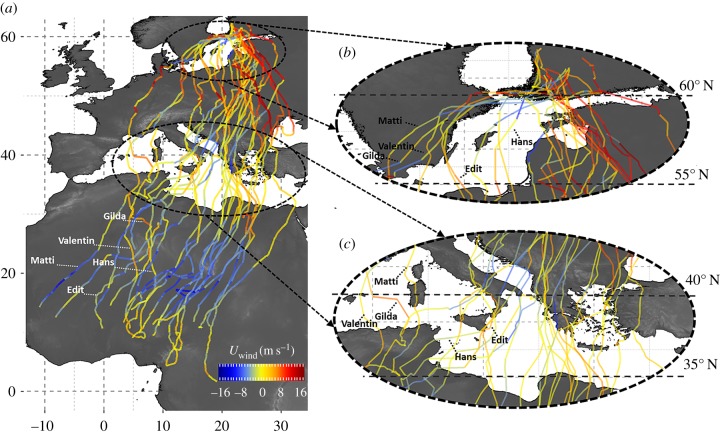
(*a*) Routes of 28 juvenile honey buzzards migrating from Finland to sub-Saharan Africa in relation to zonal wind speed (*U*_wind_, colour scale) encountered en route. Blues indicate winds with a westward component (*U*_wind_ < 0) and reds indicate winds with an eastward component (*U*_wind_ > 0). Insets zoom in on routes across (*b*) the Baltic and (*c*) the Mediterranean. Name labels highlight routes taken by five individuals that departed from Finland in a south-westward direction, through Scandinavia or across the Baltic Sea, and that survived until the end of their first outbound migration.

There was a clear positive influence of *U*_wind_ on *U*_bird_ across most of the flyway (figure 1). Eighteen of 27 individuals initiated migration in a south-eastward direction in eastward winds across the Gulf of Finland (figure 1*b*). All but two (Matti and Lisa) of nine individuals that departed westward or south-westward across Scandinavia and the Baltic Sea experienced winds with a westward component (figure 1*b*). Individuals that departed across Scandinavia continued to fly overland, even in weak sidewinds or in moderate eastward winds, until they reached southern Sweden. They then crossed the Baltic Sea in a south-eastward direction in eastward winds (e.g. Edit, figure 1*b*) or south-westward in westward winds (e.g. Gilda and Valentin, figure 1*b*). Once over mainland Europe, most birds experienced winds with a moderate to strong eastward component (figure 1*a*, 45–55° N) and concomitantly moved in a south-eastward direction. Some birds did move south-westward over Eastern Europe and the Balkans when they encountered westward winds (figure 1*a*, 40–50° N).

One bird (Julia) ended up over the Black Sea and initiated a reverse migratory movement, flying north-westward in opposing eastward winds until she approached the coast, but then continued travelling over water until near the Bosphorus. Over the Mediterranean Sea, the honey buzzards usually encountered winds with a weak zonal component (figure 1*c*). Some sea-crossing individuals engaged in pronounced longitudinal movements (e.g. Hans) that were not directed towards the nearest land. Once over Africa, most birds moved south-westward in strong westward winds (figure 1*a*). Three of four birds that travelled south-eastward over the Sahara did so in unusual eastward winds (figure 1*a*, Gilda, Hans and Valentin).

### (b) Innate and parental factors

Including a random intercept for individuals significantly improved the most parsimonious GLM (table 1, model 3) but did not significantly alter estimates for wind effects compared with model 1. We did not find evidence for parental effects by nesting individuals per territory, as indicated by the small increase in AIC and log-likelihood compared with the model including only individual (electronic supplementary material, table S4). Adding sex as a fixed categorical effect to our most parsimonious GLM revealed no significant differences between males and females and so we did not use it for multilevel modelling (*p* = 0.13).

### (c) Identifying influential geographical features

We mapped residuals from model 2 from the electronic supplementary material, table S4 (electronic supplementary material, figure S3*a*) to see when and where birds responded differently to wind while crossing geographical barriers (electronic supplementary material, figure S3*b*). The birds that departed into Scandinavia moved west faster than expected from their average individual response to wind conditions, especially the two individuals that departed westward in eastward winds (Matti and Lisa; electronic supplementary material, figure S3*b*, blues). Over the Black Sea and over the Mediterranean Sea, there occurred many ‘events’ whereby birds travelled westward (blues) or eastward (reds) faster than predicted by our most parsimonious model (e.g. Sven and Hans; electronic supplementary material, figure S2*c*). Other extremely low or high residual *U*_bird_ values occurred over Africa and across the whole journey birds tended to move westward moderately faster than predicted (electronic supplementary material, figure S2, greens), and to head downwind more over water than over land.

### (d) Influence of wind and geography on the total longitudinal displacement of birds

We distinguished between the nine birds that departed across Scandinavia or across the Baltic Sea and the 18 individuals that departed across the Gulf of Finland to predict the birds' total longitudinal displacements. However, we could not find a way to categorize individuals based on route choice in other parts of the flyway. For example, when birds arrive at the Mediterranean in roughly the same location, they continue along roughly the same flight direction under similar wind conditions.

**Figure 2.**
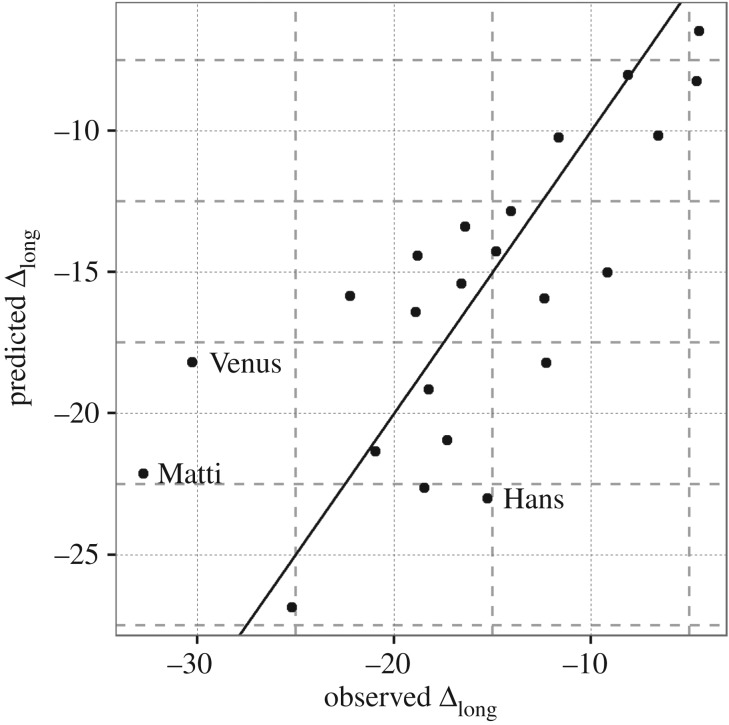
Predicted versus observed total longitudinal displacements (Δ_long_[°]) of 23 juvenile honey buzzards that survived their first autumn migration (excluding one of 24 survivors with large gaps in tracking data) based on our most parsimonious model (table 2, model 2). Points above the black line indicate cases where a bird ended up further west than predicted based on the wind conditions it encountered en route. Points below the black line are cases where birds ended up further east than predicted. Name labels indicate three individuals with relatively high standardized residual values (i.e. worst predictions).

Multiple linear regression models revealed that 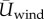 and 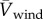 had a significant additive effect on the birds' total longitudinal displacements (Δ_long_[°], table 2). Route choice at departure, by contrast, only had a marginally significant effect on Δ_long_[°] (table 2), probably because only one bird that departed into Scandinavia in opposing winds (Matti) survived until the end of migration. Nevertheless, we found that the individuals with the largest residual longitudinal displacements (figure 2, Hans, Matti and Venus) had all engaged in longitudinal movements that were poorly accounted for by local wind conditions at some point in their journey (figure 1).

**Table 2.** Statistical summary of multiple linear regression models predicting total longitudinal displacements (Δ_long_[°]) of 23 juvenile honey buzzards that survived their first autumn migration (excluding one of 24 survivors with large gaps in tracking data). (Intercepts estimate average Δ_long_ in the absence of wind for all birds (models 1, 2 and 4) or for birds that departed Finland in a south-westward direction (models 3 and 5). Regression coefficients (*β*'s) estimate additional longitudinal displacement for every 1 m s^−1^ change in the mean *U*_wind_ (*β_U_*_wind_), in the mean *V*_wind_ (*β_V_*_wind_) and an interaction effect between the two wind components (*β_U_*_wind__:_ *_V_*_wind_). In models 3 and 5, *β*_departure_ estimates the mean difference in Δ_long_ for birds that departed in a south-eastward direction compared with those that departed in a south-westward direction. The most parsimonious model is given in italics.)

	intercept		*β_U_*_wind_		*β_V_*_wind_		*β*_departure_		*β_U_*_wind:_*_V_*_wind_		AIC	adjusted *R*^2^
	estimate	*p*-value	estimate	*p*-value	estimate	*p*-value	estimate	*p*-value	estimate	*p*-value		
model 1	−15.59	4.21 × 10^−10^	1.97	0.046							157.20	0.14
*model 2*	*−17.93*	*5.91 × 10^−12^*	*2.06*	*0.009*	*−3.01*	*0.001*					*146.13*	*0.49*
model 3	−22.08	1.60 × 10^−8^	2.69	0.002	−2.02	0.031	6.49	0.058			143.68	0.55
model 4	−17.71	1.23 × 10^−11^	2.05	0.009	−2.97	0.001			−0.72	0.180	145.90	0.51
model 5	−21.39	7.96 × 10^−8^	2.61	0.003	−2.12	0.026	5.65	0.108	−0.49	0.345	144.51	0.55

## 4. Discussion

Long-distance migrant landbirds that breed in close vicinity of each other in Europe typically spread out over vast geographical ranges in sub-Saharan Africa. Our results demonstrate that for honey buzzards, such low migratory connectivity is owing largely to stochasticity in the wind conditions that juvenile migrants encounter on their first outbound migration [4]. Although most juvenile honey buzzards travel south-eastward in eastward winds over northern and central Europe, this movement is offset by the fact that on average, juvenile honey buzzards move westward at a rate of 0.87 m s^−1^ in windless conditions, and by the prevalence of westward winds over the Sahara, which constitutes the longest segment of their journey. There were minor but significant differences in mean migratory orientation between individuals, but wind accounted for at least half the bird's total longitudinal displacement. All juveniles, both males and females, ended up further west than where they started their journey in southern Finland, and more than half ended up west of 10° E, well within the wintering range of honey buzzards that breed as far west as Sweden [24] and the Netherlands [34,37].

Atmospheric circulation patterns seem to impact on migratory connectivity in migrant landbird populations in different ways. On the one hand, stochasticity in wind conditions drives low migratory connectivity between breeding and non-breeding ranges of the Finnish honey buzzard population. On the other hand, the Afro-Palaearctic flyways are characterized by distinct latitudinal wind regimes [34] that are likely to lead to predictable patterns of migratory connectivity between different breeding populations. Juveniles that hatched in eastern breeding populations, such as those we studied here, are likely to end up in the core wintering range of conspecifics that breed in western breeding populations. By contrast, it seems unlikely that juveniles which hatched in western Europe would end up in the non-breeding range of conspecifics that breed further east and that winter in central and eastern Africa. Atmospheric circulation patterns may therefore help explain genetic structure of extant populations of *P. apivorus*, as has been done for marine migrants based on ocean currents [51].

We were unable to directly account for geography to predict the total longitudinal displacement of juvenile honey buzzards. Moreover, the honey buzzards were not as reluctant to engage in long sea-crossings as many larger soaring migrants [21,23]. Nevertheless, and contrary to our expectations, geography affected the longitude at which certain individuals settled. For example, one of two birds that departed westward from Finland into Scandinavia in opposing winds survived its first migration and ended up further west in Africa than any other juvenile (Matti). In other cases, birds ended up further west or east than predicted because they engaged in protracted downwind movements over the Mediterranean or other barriers, and because they did not compensate for these movements later in their journey (e.g. Hans and Venus). We investigated all tracks in detail to obtain clues about why these birds engaged in such movements. One bird (Sven) started flying westward when night fell during its sea-crossing, roosted on a westward-sailing ship shortly thereafter, continued flying westward in westward winds the next morning and died a few days later in northern Algeria. This bird thus probably flew downwind because it was in relatively poor condition [52,53]. The fact that another bird suddenly changed travel direction at nightfall while crossing the Black Sea suggest the birds may generally be more hesitant to fly over water at night [54,55]. However, not all sea-crossing individuals behave in the same way and it remains difficult to generalize our observations into a mechanistic, deterministic model. A simple innate migration strategy that leaves room for flexible responses to highly stochastic conditions is probably highly adaptive for migrant birds [4].

### (a) Wind, geography and mortality on the first outbound migration

The survival rate of juvenile honey buzzards was much higher than expected based on previously reported survival rates of migrating raptors in the Afro-Palaearctic flyways [56,57]. No less than 74.2% of all tagged fledglings and 88.8% of all individuals that initiated migration survived their first migration. Moreover, none of the juveniles died by drowning, in sharp contrast to the high mortality rate among larger soaring migrants that attempt long flights across the Mediterranean [23,58]. Interestingly, all three juveniles that died during the first autumn migration (F2, F3, M4) had left Finland through Scandinavia or around the Baltic Sea. However, the circumstances under which these birds were lost suggest they died owing to different causes and we do not think mortality is systematically higher along this flyway [59].

### (b) Potential carry-over effects of early-life migration experiences

Depending on where juvenile honey buzzards end up settling at non-breeding sites, they may learn very different spring migration routes when they first return to Europe as immature birds. Honey buzzards usually spend at least one whole year in sub-Saharan Africa before they first return to Europe, and they may move further east or west before their first spring migration [60]. Of the individuals we studied here, 12 reached their third calendar year. All of these moved over long distances within Africa, but most returned to a point near the location or region as where they first settled for long periods, and initiated spring migration from there (electronic supplementary material, figure S1). Assuming that immature honey buzzards rely on a simple innate migration programme on their first return migration, we would then expect individuals that settled non-breeding grounds in West Africa to take a western route on their first return migration. However, at some point, immature honey buzzards migrate at the same time as adults from whom they then learn complex detours through the wind regimes and around the geographical barriers that characterize the African-Eurasian flyways [24,34]. By now, the juveniles we tracked have all died or reached adulthood, and we are working to determine how many birds eventually manage to learn the traditional detours, at what age learning takes place and how early-life experiences such as those described in this paper ultimately affect development of individual migration routes and natal dispersal.

### (c) Implications for other migrants

We expect that wind conditions and geography will shape the first outbound migrations and non-breeding distributions of many other migrant landbirds in similar ways as we described here, at least for species that rely on a simple innate vector-based navigation during their first migration. It is possible that the influence of wind is relatively more important in the Afro-Palaearctic flyways compared with, for example, the Americas, where geography strongly affects migratory connectivity [1,2]. If so, we would also expect that there is stronger selection for stochastic rather than deterministic (cf. [27]) migration tactics in the Old World compared with the New World, because in the latter, breeding and non-breeding grounds are connected only by a narrow land mass, whereas in the former, metaphorically speaking, ‘all flyways lead to Rome’. However, theoretical studies have shown that a high within-clutch variability of innate migratory headings is also advantageous for migrants breeding in North America, because it increases the likelihood that at least one chick will be able to contend with stochastic atmospheric conditions [61]. Our results suggest that pairs of long-lived honey buzzards with small clutch size (2 eggs) benefit from a similar bet-hedging strategy across multiple years.

There are migrant landbirds in the Old World that learn complex detours without adult guidance that are not necessarily optimal with respect to seasonal winds [62], often to reach a specific non-breeding area [26]. It remains unclear, however, why and how such a high degree of migratory specialization is maintained. Red-backed shrikes *Lanius collurio*, for example, appear to back-track the routes along which ancestors colonized Iberia from East Europe and Africa [62], while other migrant birds have developed innovative migration strategies over much shorter time scales after colonizing new breeding areas [63]. It could be that certain innate or environmental factors constrain the ability of juveniles of conservative migrants to learn alternative strategies, and lifelong tracking studies will be crucial to understand under what conditions bet-hedging and deterministic migration strategies ultimately emerge. This matters also for conservation, because breeding populations of migrant landbirds like honey buzzards are unlikely to be conserved through protected areas but rather by innovative landscape-based conservation approaches on the wintering grounds [4,64,65].

Non-breeding distributions of migrant birds are strongly determined by connectivity to breeding areas [66] and evolution of migratory links depends on the distance birds have to travel over barriers [67]. Atmospheric circulation patterns can strongly impact on this connectivity, as favourable winds can turn a formidable barrier into a freeway for migrant birds, and vice versa [34,68]. Models that simulate juvenile migrations of birds show that it is possible for birds to reach their non-breeding areas through real-life wind fields using simple vector-based orientation [25]. Similarly, we can replicate the distribution of non-breeding adult sea-turtles and eel by modelling drift trajectories of their hatchlings or larvae through extant ocean currents [69–71]. This suggests that non-deterministic, go-with-the-flow migration strategies are highly adaptive in the juvenile life-stage of many flying and swimming organisms, even if they need to compensate for the drift they accumulated as juveniles to return to their natal site in a later stage of life [72,73].

## Supplementary Material

Figure S1

## Supplementary Material

Figure S2

## Supplementary Material

Figure S3

## Supplementary Material

Table S1

## Supplementary Material

Table S2

## Supplementary Material

Table S3

## Supplementary Material

Table S4

## Data accessibility

The processed and wind-annotated satellite-tracking data and code needed for reproducing our analyses are made through the Dryad repository: http://dx.doi.org/10.5061/dryad.6nj94 [74]. The global shoreline database used for mapping is publically accessible via https://www.ngdc.noaa.gov/mgg/shorelines/gshhs.html.
