# Supplementary material for: Wind conditions and geography shape the first outbound migration of juvenile honey buzzards and their distribution across sub-Saharan Africa
Source: Proc Biol Sci. 2017 May 24;284(1855):20170387. doi: 10.1098/rspb.2017.0387 (PMC5454264; doi:10.1098/rspb.2017.0387)
Supplement: Figure S1 [file rspb20170387supp1.pdf]

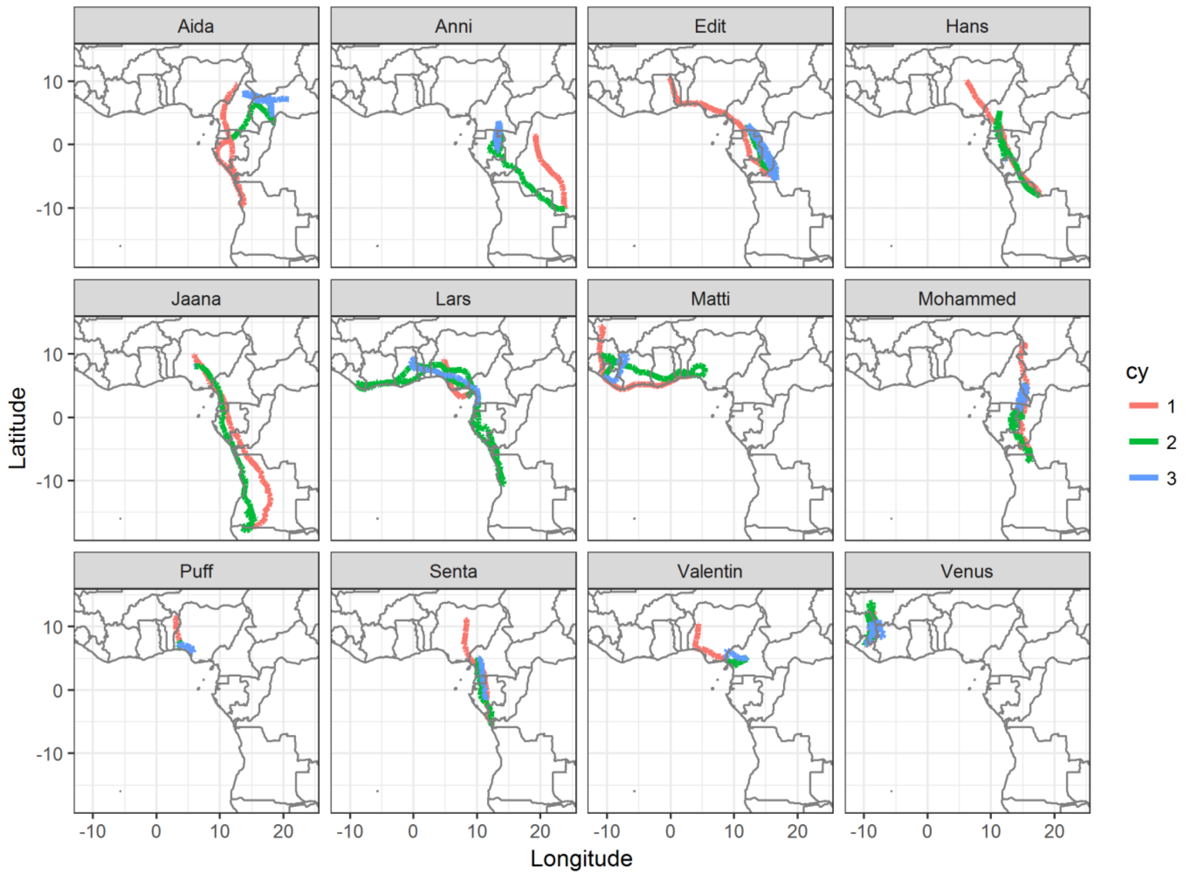

Figure S1. Intra-African movements from the end of autumn migration until the start of adult spring migration for twelve honey buzzards that survived until at least their third calendar year.

Honey buzzards engage in pre-migratory movements in Europe and also make itinerant movements within sub-Saharan Africa during the non-breeding season. We therefore have to develop some simple rules on the basis of which to categorize the migration period. Following procedures from previous honey buzzard studies (Vansteelant et al., 2015; Vansteelant et al., 2017), we used the ´deg.dist()´ function from the ‘fossil’ package (Vavrek, 2011) in R v3.3.0 (R Core Development Team, 2013) to calculate the great-circle distance between the first and the last fix obtained on each day. Based on the bimodal distribution of daily travel distances we then distinguished days with long-distance movements (> 25km between roosts) from days with local movements (< 25km between roosts) and partitioned each track into uninterrupted segments of directed and local movement. As long as a bird made local movements for at least three consecutive days in the breeding range (north of 58°N) we assumed it was engaging in pre-migratory movements. Migration started on the first day after the last segment of pre-migratory movement. We assumed migration had ended as soon as a bird engaged in local movements for at least three consecutive days in the wintering range (south of 15°N).

According to this classification individuals still displace substantially further south, and sometimes east or west, during their first calendar year after completing the main stage of migration. However,

most individuals return to or near the location where they ended their first autumn during the 2nd CY or later, before initiating their first spring migration*. The main exceptions, in which case birds established non-breeding areas considerably further east or west are Aida, Anni, Edit and Valentin. But these birds still returned to generally the same region as where they originally ended their first autumn migration. While undertaking intra-African movements honey buzzards are likely searching for food, possibly riding the seasonal green wave that moves through sub-Saharan Africa, and less affected by regional wind conditions. The ecology of these movements is therefore distinct from the ecology of the migratory movement between Europe and Africa.

**not all 12 individuals initiated their first spring migration in the 3CY but only the 4CY. However, we only show data until the 3CY for legibility of the figures and because intra-African movements seem to be repeated between known locations after that (Byholm, unpubl. data.).*
