# Supplementary material for: Wind conditions and geography shape the first outbound migration of juvenile honey buzzards and their distribution across sub-Saharan Africa
Source: Proc Biol Sci. 2017 May 24;284(1855):20170387. doi: 10.1098/rspb.2017.0387 (PMC5454264; doi:10.1098/rspb.2017.0387)
Supplement: Figure S2 [file rspb20170387supp2.pdf]

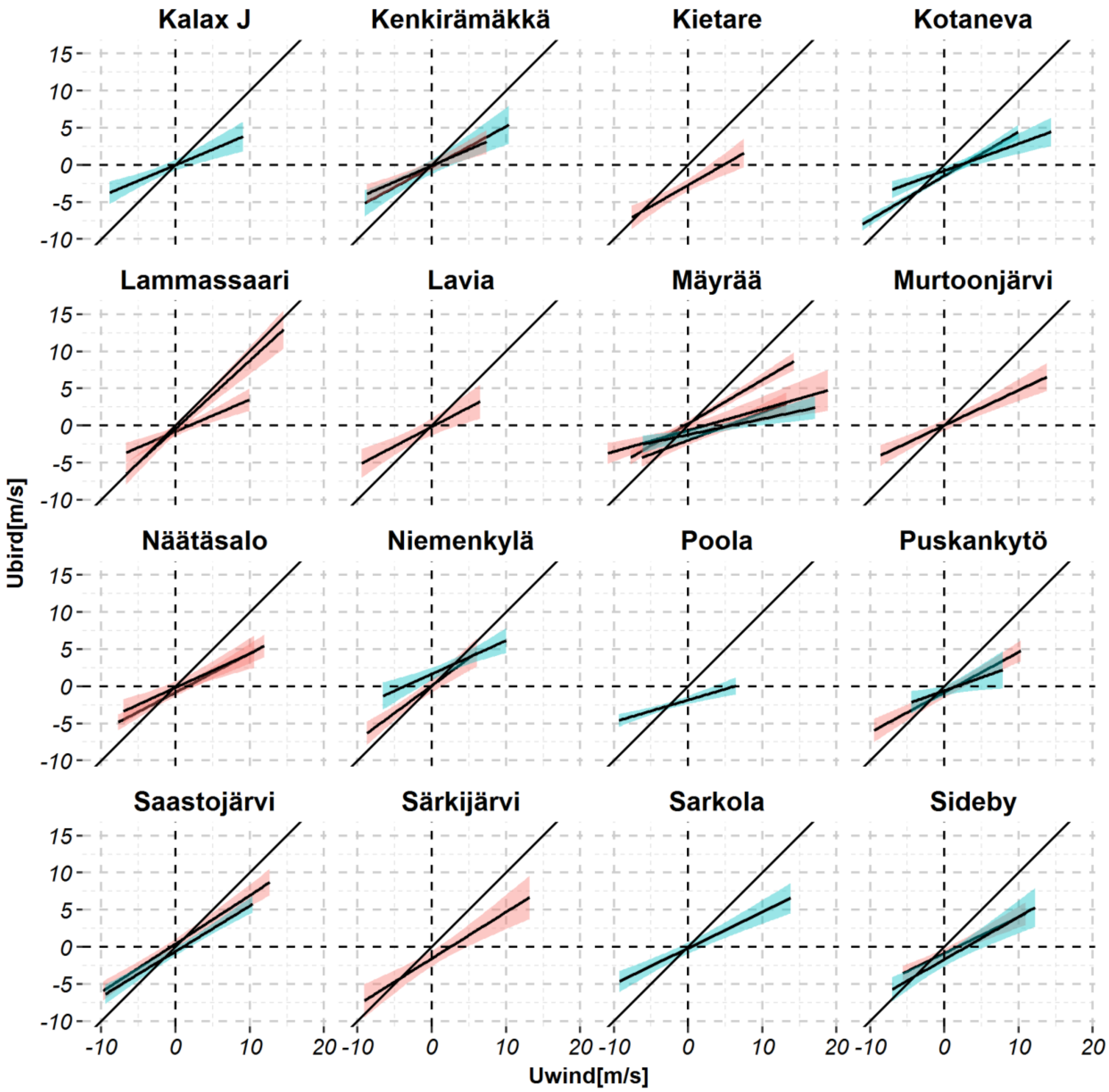

Fig S2. Linear relationship between U_{bird} and U_{wind} per individual (graphs), showing all females (pink) and males (blue) per territory. Dashed lines indicate main axes and solid line indicates what would be a perfect linear fit between U_{bird} and U_{wind}. Generally all birds show a similar response to U_{wind}, although some birds have intercepts that differ from 0, which is what we would expect if birds vary in terms of innate migratory headings. In some birds the effect of sidewinds (i.e. the slope) seems to be greater than for others, but it is not immediately clear what individual differences would account for such a difference. Juveniles that originated from the same territory do not seem to differ in a consistent way from individuals on other territories, i.e. siblings do not seem to behave more similar than other juveniles.
