# Supplementary material for: Wind conditions and geography shape the first outbound migration of juvenile honey buzzards and their distribution across sub-Saharan Africa
Source: Proc Biol Sci. 2017 May 24;284(1855):20170387. doi: 10.1098/rspb.2017.0387 (PMC5454264; doi:10.1098/rspb.2017.0387)
Supplement: Figure S3 [file rspb20170387supp3.pdf]

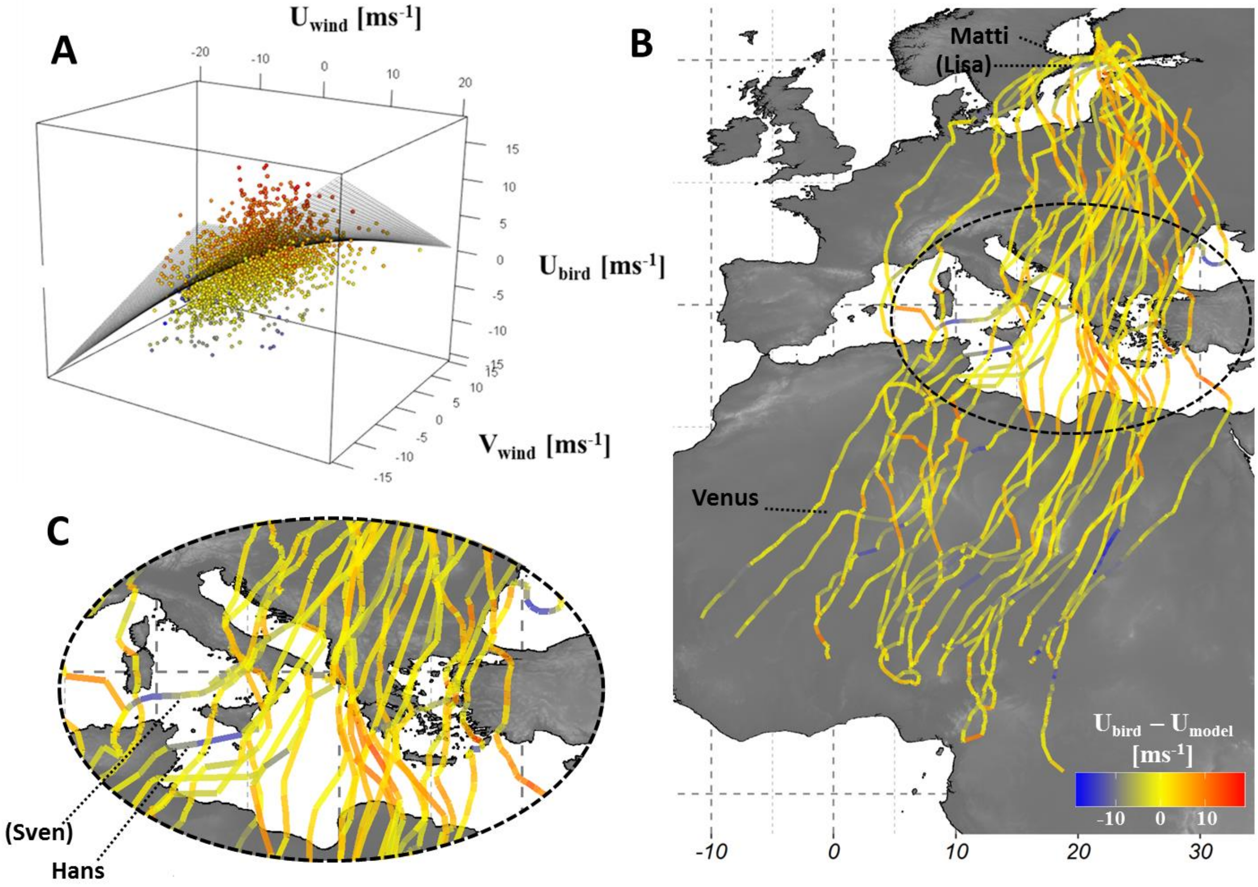

Figure S3 Visualizing when and where birds deviate from their average individual response to wind. (A) 3D-plot showing hourly longitudinal bird speed (U_{bird}) in relation to U_{wind} and V_{wind} as measured by satellite tracking (dots) and the response (grey surface) predicted for ´Lars´ by our most parsimonious mixed effects model (Table S4, model 2). Measured speeds were colour coded according to residual hourly longitudinal speed (U_{bird} - U_{model}). (B) We then mapped residual longitudinal speeds to determine when and where juvenile honey buzzards moved westward (blue) or eastward (red) faster than predicted by local wind conditions. (C) Name labels indicate pronounced but poorly predicted longitudinal movements of which also the total longitudinal displacements were poorly predicted (Fig3, Hans, Matti & Venus). Name labels between brackets indicate movements that were also poorly predicted, but that were made by two birds that died before the end of migration (Sven & Lisa) and which were not considered for modelling the birds´ total longitudinal displacements. Inset highlights the Mediterranean as much of the most deviant behaviour occurred there.
