# Supplementary material for: Wind conditions and geography shape the first outbound migration of juvenile honey buzzards and their distribution across sub-Saharan Africa
Source: Proc Biol Sci. 2017 May 24;284(1855):20170387. doi: 10.1098/rspb.2017.0387 (PMC5454264; doi:10.1098/rspb.2017.0387)
Supplement: Table S1 [file rspb20170387supp4.pdf]

Bird	Nick name	Device	Sex	Territory	Tag date	Death/Tag failure	
						Date	Context
F1	*Elisabeth*	*118750*	*f*	*Kenkirämäkkä*	*06/08/2012*	*Aug-12*	*Predated on nest*
F2	Lisa	118749	f	Näätäsalo	02/08/2013	11/10/2013	Never left roost
F3	Emma	HBFI03	f	Lammassaari	05/08/2013	14/10/2013	Stop transmission
F4	Roosa	59612	f	Mäyrää	05/08/2011	09/02/2012	Stop transmission
F5	Gilda	59956	f	Mäyrää	08/08/2012	09/11/2012	Corpse retrieved
F6	Kirsi	118748	f	Sideby	10/08/2012	13/11/2012	Corpse retrieved
F7	Puff	59957	f	Kenkirämäkkä	06/08/2011	14/05/2013	Never left roost
F8	Ulla	HBFI01	f	Lammassaari	05/08/2013	09/12/2013	Stop transmission
F9	Ella	59956	f	Saastojärvi	13/08/2013	16/12/2013	Stop transmission
F10	Julia	HBFI02	f	Murtoonjärvi	16/08/2013	13/01/2014	Stop transmission
F11	Aida	118746	f	Puskankytö	02/08/2012	18/07/2014	Corpse retrieved
F12	Heidi	118748	f	Niemenkylä	08/08/2013	20/12/2014	Stop transmission
F13	Venus	59964	f	Kietare	08/08/2012	12/05/2015	Never left roost
F14	Jaana	59804	f	Lavia	28/07/2011	13/05/2015	Corpse retrieved
F15	Edit	HBFI08	f	Näätäsalo	02/08/2013	18/06/2015	Stop transmission
F16	Anni	HBFI07	f	Mäyrää	05/08/2013	21/07/2016	Stop transmission
F17	Senta	59955	f	Särkijärvi	08/08/2012	alive	-
M1	*Clark*	*118749*	*m*	*Kietare*	*08/08/2012*	*Aug-12*	*Died on nest*
M2	*Hannu*	*59954*	*m*	*Kenkirämäkkä*	*06/08/2012*	*Aug-12*	*Predated on nest*
M3	*Ivar*	*118750*	*m*	*Sideby*	*06/08/2013*	*21/09/2013*	*Stop transmission*
M4	Sven	223	m	Sideby	06/08/2013	06/10/2013	Stop transmission
M5	Miikka	HBFI06	m	Niemenkylä	08/08/2013	04/04/2014	Large gaps in data
M6	Piff	59644	m	Kenkirämäkkä	06/08/2011	18/02/2012	Stop transmission
M7	Tor	59806	m	Sarkola	28/07/2012	29/12/2012	Stop transmission
M8	Rudolf	59358	m	Kotaneva	02/08/2012	31/01/2013	Stop transmission
M9	Hans	59963	m	Puskankytö	02/08/2012	22/03/2014	Stop transmission
M10	Viljo	400	m	Poola	02/08/2014	26/10/2014	Stop transmission
M11	Matti	HBFI05	m	Kotaneva	09/08/2013	28/05/2015	Never left roost
M12	Lars	59805	m	Kalax J	08/08/2011	04/10/2015	Stop transmission
M13	Valentin	118747	m	Mäyrää	08/08/2012	09/03/2016	Stop transmission
M14	Mohammed	HBFI04	m	Saastojärvi	13/08/2013	alive	-

Table S1 Metadata of 17 female and 14 male juvenile honey buzzards satellite-tagged as fledglings in southern Finland. Individuals highlighted in italics died before or just after initiating their first autumn migration and were not used in our analyses. We used all remaining 27 birds to model hourly longitudinal bird speeds (U_{bird}) as a function of the hourly zonal wind component (U_{wind}) and latitudinal wind component (V_{wind}) encountered en route. Three more birds died before completing migration and tracking data for one bird had large gaps (M5). Therefore, we only used the remaining 23 survivors highlighted in light grey to model the total longitudinal shift (Δ_{long} [°]) of juvenile honey buzzards between the start and the end of their migratory journey.
