# Supplementary material for: Wind conditions and geography shape the first outbound migration of juvenile honey buzzards and their distribution across sub-Saharan Africa
Source: Proc Biol Sci. 2017 May 24;284(1855):20170387. doi: 10.1098/rspb.2017.0387 (PMC5454264; doi:10.1098/rspb.2017.0387)
Supplement: Table S2 [file rspb20170387supp5.pdf]

Nick name	N	U_{bird} [ms^{-1}]				U_{wind} [ms^{-1}]				V_{wind} [ms^{-1}]			
		**mean**	**SE**	**min**	**max**	**mean**	**SE**	**min**	**max**	**mean**	**SE**	**min**	**max**
Aida	74	-0.24	0.48	-12.12	9.21	1.06	0.61	-9.47	10.30	-0.24	0.51	-8.63	12.49
Anni	180	1.01	0.35	-10.26	15.16	1.25	0.43	-7.74	14.27	-2.35	0.35	-13.20	9.33
Edit	160	-0.75	0.31	-12.41	6.82	0.11	0.33	-7.71	11.98	-0.76	0.34	-11.31	8.71
Ella	99	0.01	0.49	-10.28	15.81	-0.67	0.55	-9.78	12.70	-3.00	0.43	-13.79	7.31
Emma	114	-0.33	0.35	-11.26	7.22	1.04	0.37	-6.68	9.93	1.40	0.38	-5.04	11.44
Gilda	95	-1.09	0.32	-9.02	8.77	2.34	0.45	-6.21	13.26	0.89	0.48	-10.33	11.24
Hans	71	-0.61	0.38	-14.87	5.94	-0.08	0.29	-4.45	7.87	1.63	0.35	-8.70	9.29
Heidi	60	-1.49	0.56	-13.21	7.96	-2.18	0.52	-8.71	6.08	-2.71	0.50	-12.36	4.41
Jaana	42	-1.33	0.61	-12.27	7.36	-2.28	0.68	-9.44	6.52	0.00	0.45	-4.87	9.59
Julia	138	0.27	0.43	-12.54	13.33	0.47	0.48	-8.59	13.78	-3.48	0.39	-11.86	9.76
Kirsi	65	-0.65	0.42	-8.87	7.63	0.37	0.58	-5.61	10.93	0.35	0.44	-5.90	13.28
Lars	81	-0.58	0.39	-9.13	11.04	-1.38	0.44	-8.88	9.07	0.06	0.33	-6.65	9.22
Lisa	61	-0.06	0.49	-11.34	8.90	0.21	0.56	-7.02	10.57	0.94	0.52	-9.59	8.83
Matti	208	-2.39	0.25	-9.93	8.68	-1.59	0.30	-11.04	9.95	0.30	0.19	-7.04	7.92
Miikka	98	2.62	0.40	-7.44	14.18	2.13	0.39	-6.60	10.02	-1.70	0.40	-8.56	7.99
Mohammed	174	0.24	0.37	-11.22	10.92	1.33	0.39	-9.44	10.40	-2.36	0.23	-8.99	4.12
Piff	44	-1.05	0.59	-8.93	6.46	-1.46	0.69	-9.03	10.40	-0.59	0.60	-7.59	9.42
Puff	51	-0.82	0.39	-5.84	7.35	-1.68	0.55	-8.76	7.34	-1.65	0.39	-7.17	8.80
Roosa	57	-1.06	0.41	-7.41	4.79	-1.51	0.73	-10.82	18.85	0.12	0.42	-5.31	8.31
Rudolf	87	-0.59	0.34	-7.89	9.99	0.48	0.48	-7.03	14.38	-0.33	0.37	-10.08	10.38
Senta	43	-1.36	0.70	-10.44	7.95	0.29	0.76	-9.12	13.14	-1.31	0.42	-5.58	4.89
Sven	74	-1.75	0.55	-13.98	6.74	-0.13	0.54	-7.01	12.23	-1.37	0.64	-10.85	10.64
Tor	45	-0.32	0.56	-8.36	9.68	-0.35	0.86	-9.25	13.82	-1.92	0.48	-9.70	5.41
Ulla	151	0.05	0.49	-12.77	13.45	0.47	0.34	-6.67	14.56	-2.24	0.30	-10.14	9.36
Valentin	85	-0.47	0.27	-6.19	4.79	3.46	0.50	-6.12	17.12	1.21	0.48	-9.50	11.17
Venus	56	-3.29	0.49	-13.89	5.77	-1.03	0.52	-7.58	7.54	-0.62	0.39	-6.22	6.70
Viljo	129	-2.70	0.24	-9.46	4.05	-2.92	0.34	-9.32	6.41	0.96	0.24	-3.54	9.25

Table S2 Summary statistics for 27 birds that were used for modelling hourly longitudinal bird speed, showing sample size (N; i.e. number of GPS-fixes) as well as the mean, standard error (SE), minimum and maximum hourly longitudinal bird speed (U_{bird}), hourly zonal wind speed (U_{wind}) and hourly latitudinal wind speed (V_{wind}) per bird.
