# Supplementary material for: Wind conditions and geography shape the first outbound migration of juvenile honey buzzards and their distribution across sub-Saharan Africa
Source: Proc Biol Sci. 2017 May 24;284(1855):20170387. doi: 10.1098/rspb.2017.0387 (PMC5454264; doi:10.1098/rspb.2017.0387)
Supplement: Table S3 [file rspb20170387supp6.pdf]

Nick name	Start of migration	End of migration	Duration [days]	Δ_{long} [°]	Departure direction
Aida	09/09/2012	14/10/2012	35	-9.16	S-SE
Anni	14/09/2013	19/10/2013	35	-4.62	S-SE
Edit	18/09/2013	29/10/2013	41	-16.57	W-SW
Ella	21/09/2013	24/10/2013	33	-11.64	S-SE
Gilda	15/09/2012	05/11/2012	51	-22.23	W-SW
Hans	22/09/2012	06/11/2012	45	-15.26	W-SW
Heidi	26/09/2013	22/10/2013	26	-14.81	S-SE
Jaana	11/09/2011	17/10/2011	36	-18.47	S-SE
Julia	20/09/2013	10/10/2013	20	-4.51	S-SE
Kirsi	13/09/2012	28/10/2012	45	-12.26	S-SE
Lars	15/09/2011	27/10/2011	42	-17.28	S-SE
Matti	10/09/2013	24/10/2013	44	-32.75	W-SW
Mohammed	07/09/2013	07/10/2013	30	-8.11	S-SE
Piff	15/09/2011	11/10/2011	26	-18.23	S-SE
Puff	16/09/2011	17/10/2011	31	-18.91	S-SE
Roosa	14/09/2011	08/10/2011	24	-20.93	S-SE
Rudolf	16/09/2012	03/11/2012	48	-12.37	S-SE
Senta	21/09/2012	19/10/2012	28	-16.38	S-SE
Tor	06/09/2012	08/10/2012	32	-14.07	S-SE
Ulla	14/09/2013	11/10/2013	27	-6.57	S-SE
Valentin	15/09/2012	05/11/2012	51	-18.80	W-SW
Venus	21/09/2012	25/10/2012	34	-30.24	S-SE
Viljo	24/09/2014	26/10/2014	32	-25.17	S-SE

Table S3 Timing, duration, total longitudinal displacement (Δ_{long} [°]) and departure direction from Finland of 23 individuals that survived their first autumn migration and used for modelling Δ_{long} as a function of mean wind conditions encountered en route and route choice at departure (excluding one of 24 survivors with large gaps in tracking data).
