# Supplementary material for: Wind conditions and geography shape the first outbound migration of juvenile honey buzzards and their distribution across sub-Saharan Africa
Source: Proc Biol Sci. 2017 May 24;284(1855):20170387. doi: 10.1098/rspb.2017.0387 (PMC5454264; doi:10.1098/rspb.2017.0387)
Supplement: Table S4 [file rspb20170387supp7.pdf]

	Model 1		Model 2		Model 3	
**AIC**	13340.34		13275.54		13277.54	
**LogLik**	-6665.168		-6631.769		-6631.769	
**DF**	2538		2512		2512	
**Fixed effects**						
Intercept	-0.759	(-0.067)	-0.73	(-0.156)	-0.729	(-0.156)
U_{bird}	0.468	(-0.015)	0.465	(-0.015)	0.465	(-0.015)
V_{bird}	-0.111	(-0.017)	-0.087	(-0.018)	-0.087	(-0.018)
$U_{bird}:V_{bird}$	-0.026	(-0.003)	-0.025	(-0.003)	-0.025	(-0.003)
**Random effects**						
individual	-	-	0.722	(-3.245)	0.722	(-3.245)
territory	-	-	-	-	0.001	-

Table S4 Modelling individual and parental effects. We compare the wind effects model (model 1) to a mixed linear effect model including a random effect for variable intercepts per individual (model 2) and to a nested mixed linear effect model accounting for variable intercepts per individual within each territory (model 3). For each model we report AIC, log likelihood values and degrees of freedom. In addition, we show the estimates (SE) of all fixed and random effects in each model. An ANOVA revealed model 2 was significantly better than model 1 ($p < 0.001$), which is also reflected in the lower AIC and Log Likelihood values. There was, however, no evidence for differences in wind drift patterns between individuals from different territories. Accounting for differences in intercepts between individuals in model 2 does not significantly alter estimates for wind effects compared to model 1, and overall U_{bird} varies much more strongly with wind conditions than it does between individuals.
